# The synaptotagmin juxtamembrane domain is involved in neuroexocytosis

**DOI:** 10.1016/j.fob.2015.04.013

**Published:** 2015-04-30

**Authors:** Paola Caccin, Michele Scorzeto, Nunzio Damiano, Oriano Marin, Aram Megighian, Cesare Montecucco

**Affiliations:** a Dipartimento di Scienze Biomediche, Università di Padova, Via Ugo Bassi 58/B, 35131 Padova, Italy; b Institute for Neuroscience, National Research Council, Via Ugo Bassi 58/B, 35131 Padova, Italy; c CRIBI Biotechnology Centre, Via Ugo Bassi 58/B, 35131 Padova, Italy

**Keywords:** SV, synaptic vesicles, PM, presynaptic membrane, α-BTX, alpha-bungarotoxin, Syt, synaptotagmin, TM, transmembrane, NMJ, neuromuscular junction, JMS, juxtamembrane segment, h-JMS, hexyl juxtamembrane segment, h-sJMS, hexyl scrambled juxtamembrane segment, h-FJMS, hexyl fluorescent juxtamembrane segment, Synaptotagmin, Juxtamembrane domain, Anionic phospholipids, Neuromuscular junction, Neuroexocytosis

## Abstract

Synaptotagmin is a synaptic vesicle membrane protein which changes conformation upon Ca^2+^ binding and triggers the fast neuroexocytosis that takes place at synapses. We have synthesized a series of peptides corresponding to the sequence of the cytosolic juxtamembrane domain of synaptotagmin, which is highly conserved among different isoforms and animal species, with or without either a hexyl hydrophobic chain or the hexyl group plus a fluorescein moiety. We show that these peptides inhibit neurotransmitter release, that they localize on the presynaptic membrane of the motor axon terminal at the neuromuscular junction and that they bind monophosphoinositides in a Ca^2+^-independent manner. Based on these findings, we propose that the juxtamembrane cytosolic domain of synaptotagmin binds the cytosolic layer of the presynaptic membrane at rest. This binding brings synaptic vesicles and plasma membrane in a very close apposition, favouring the formation of hemifusion intermediates that enable rapid vesicle fusion.

## Highlights

• The highly cationic juxtamembrane segment of synaptotagmin juxtamembrane domain was synthesized.
• This peptide inhibits neurotransmitter release at the neuromuscular junction of mice and *Drosophila*.
• This peptide localizes mainly on the presynaptic membrane.
• The synaptotagmin juxtamembrane peptide binds monophosphoinositides in a Ca^2+^-independent manner.
• The juxtamembrane segment of synaptotagmin may contribute to the formation of the hemifusion intermediate.

## 1 Introduction

Neuroexocytosis is a central function of physiology and behavior and it consists of the Ca^2+^-regulated fusion of cytosolic neurotransmitter/neuropeptide containing vesicles (SV) with the presynaptic membrane (PM) with release of its content into the synaptic cleft. Neuroexocytosis is mediated by a nanomachine that includes components located on the SV membrane, in the cytosol and on the PM cytosolic face [1–3]. SNAP-25 and syntaxin project their protein mass into the cytosol and can form a coiled–coil complex with a SV protein termed VAMP/synaptobrevin. This heterotrimeric oligomer is termed SNARE complex [4] and the determination of its structure has indicated that SNARE complex formation occurs via coil-coiling of a ∼60 residues long SNARE domain present in the sequence of VAMP, SNAP-25 and syntaxin [5]. Other proteins play a major role in SV docking to the PM, but the SNARE complex and the SV Ca^2+^ binding protein synaptotagmin are those involved in the membrane fusion process which ultimately leads to the release of the SV content [6].

There is evidence that three or more SNARE complexes are required for neuroexocytosis to occur, i.e. a supercomplex of SNARE complexes is required [7–14]. The number of SNARE complexes present in such a nanomachine has not yet been determined with certainty and different events of exocytosis may require SNARE supercomplexes of different stoichiometries. Two cytosolic proteins, Munc-18 and complexin, are essential for the correct assembly of the neuroexocytosis nanomachine by regulating different stages of the formation of the SNARE complex and supercomplex [6]. A tentative arrangement of the supercomplex required for ultrafast neuroexocytosis has been proposed [3].

**Fig. 1:**
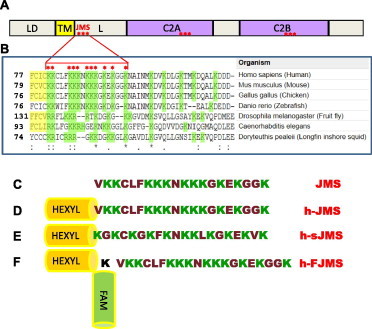
Juxtamembrane polybasic peptide in synaptotagmin sequences. (A) Schematic representation of synaptotagmin domains. LD: lumenal domain; TM: transmembrane domain; L: linker domain; C2A and B: calcium binding domains; JMS: juxtamembrane Segment. The length of each box is proportional to the number of AA in the real sequence. Red asterisks indicate clusters of positive residues. (B) Sequence alignment of synaptotagmin-1 juxtamembrane segment in different species, obtained from www.uniprot.org; yellow indicates transmembrane domain, green basic residues. (C–F) Peptides used in this study. (C) Peptide corresponding to synaptotagmin polybasic segment (JMS, residues 80-98 in mouse sequence); (D) Modified peptide, with the hexyl moiety (h-JMS); (E) Scrambled peptide (h-sJMS); (F) Fluorescent peptide (h-FJMS).

Synaptotagmins (Syt) form a large family of proteins which basically include a intravesicular domain of varying size, a transmembrane domain (TM) and a linker segment that connects the TM to two consecutive C2 domains that are exposed to the cytosol [15–17] (Fig. 1). Syt binds Ca^2+^ via the C2 domains each of which contains a conserved polycationic segment that binds anionic PM phospholipids, including phosphatidylserine and phosphatidylinositol-4,5-biphosphate, in a Ca^2+^ dependent way, leading to membrane penetration and induction of a positive curvature in the PM [18–25]. This interaction leads to membrane penetration of segments of the C2 domain and to the bridging of the SV and PM membranes [26,27]. There is a growing consensus on the possibility that Ca^2+^ and anionic phospholipid binding to synaptotagmin triggers its rapid conformational change that is transmitted to the SNARE supercomplex leading to SV-PM fusion with the delivery of the vesicle content to the post-synaptic cell [2,3,6,17,18,21–23,28]. In the case of the neuromuscular junction synapse (NMJ), which is studied here, the released neurotransmitter is acetylcholine that migrates across the intersynaptic space to induce the opening of the acetylcholine receptor channel [29–31].

Here, we have attempted to determine the possible role in neuroexocytosis of the highly cationic juxtamembrane segment of Syt by testing the effect of corresponding peptides on neurotransmitter release at the mouse and *Drosophila melanogaster* NMJ. Our approach is based on the known property of highly cationic peptides to cross the plasma membrane of cells [32–34]. We found that these peptides induce a rapid neuroparalysis, bind phosphoinositides in a Ca^2+^ independent mode and largely localize on the presynaptic membrane. Based on these findings, we propose a possible activity of the juxtamembrane segment of Syt in neuroexocytosis.

## 2 Results

### 2.1 Juxtamembrane polybasic peptides of synaptotagmin are powerful inhibitors of neuroexocytosis in the insect and mammalian synapses

Fig. 1 shows that the juxtamembrane segment of Syt 1 contains a set of positively charged residues that has the potential to interact with anionic phospholipids. This aminoacid sequence is highly conserved among the Syts involved in neuroexocytosis and among different species suggesting that it plays an important role in neuroexocytosis [16]. The NMJ also expresses Syt2 [35], but the juxtamembrane segment of this Syt isoform contains three cysteine residues that generate all kind of artifacts owing to the possibility of generating intra and inter disulfide bonds.

The peptide corresponding to the juxtamembrane segment (JMS) of the mouse neuronal Syt isoform 1 (segment 80–98 of the mouse sequence) was synthesized with or without an hexyl chain at its N-terminus to mimic the TM binding to the lipid bilayer (lower part of Fig. 1), also on the basis of a previous study where the TM domains of VAMP/synaptobrevin and of syntaxin where replaced by hydrocarbon chains [36]. A peptide with a scrambled distribution of the same residues along the sequence was also prepared to be used as a control.

**Fig. 2:**
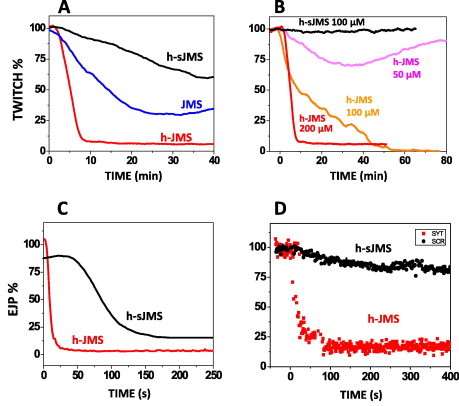
Inhibition of the neuromuscular junction induced by synaptotagmin-derived peptides. (A and B) normalized muscular twitch of mouse hemidiaphragm preparations after the addition, at time 0, of the indicated peptides. (A) Red trace: 200 μM hexyl-JMS (h-JMS), blue trace: 200 μM of the same peptide but without the hexyl hydrocarbon chain (JMS); black trace: 200 μM hexyl-scrambled sequence (h-sJMS). (B) Black trace: 100 μM scrambled hexyl peptide (h-sJMS); purple trace: 50 μM h-JMS; orange trace: 100 μM h-JMS; red trace: 200 μM h-JMS as in A, for comparison. (C) Percentage reduction of EJPs, intracellularly recorded from single mouse diaphragm muscle fibers, after adding the specific peptide to the bath. Red trace: 100 μm h-JMS, black trace: 100 μM h-sJMS. (D) Percentage reduction of EJPs, intracellularly recorded from *Drosophila melanogaster* 3rd instar larval muscles 6/7. The indicated peptides were added at time 0; red trace: 200 μm h-JMS, black trace: 200 μM h-sJMS.

The effect of the peptides was first tested in the mouse phrenic hemidiaphragm nerve preparation, a well established model of *ex vivo* neuromuscular junction (NMJ). Both the JMS peptide and its hexyl derivative (h-JMS) are able to inhibit the nerve-stimulated contraction of the muscle. The JMS peptide is a less effective inhibitor of muscle twitching that its hexylated counterpart, as expected on the basis of the higher membrane partition of the latter compound (Fig. 2A). We have used up to 0.2 mM concentration only in the hemidiaphragm NMJ; this preparations is >99% muscle volume and only a minor fraction is nerve terminals. In fact, the end plate is less than 0.1% of the total muscle surface [37]. Accordingly, the predominant muscle will take up peptide and therefore the effective amount of peptide available for the nerve terminal is greatly reduced. At the end of each experiment the integrity of the muscle was tested and the peptides were found to have no effect on muscle contraction.

The hexyl scrambled peptide (h-sJMS) has a residual effect, which can be attributed to the high number of cationic residues that inevitably makes it partially similar to the native segment in terms of charge distribution. This finding indicates that electrostatic interactions play an important, but not unique, role in the inhibitory effect. However, at lower concentrations (below 100 μM), h-sJMS is without effect (black line in Fig. 2B), whereas the native sequence still induces a defined reduction of muscular twitch. The scrambled peptide was designed by the software ‘PepControls’ (http://bioware.ucd.ie) and retains the same charge as the sequence from the synaptotagmin one. It seems very unlikely that the relative positions of the charged residue in the sequence of h-JMS and h-sJMS would alter their permeability to membranes. In fact the peptide chain and its lateral chains are flexible and the membrane permeating species is a complex among the cationic peptide and the anionic plasma membrane lipid. Thus it is safe to assume that the relative lack of effects of the control peptide h-sJMS are not a consequence of it having an unexpected low rate of entry into cells. Likewise, h-JMS and h-sJMS will have the same general effect on membrane surface potential as the total number of electric charges is the same.

In order to have a different read-out of the effect of the h-JMS peptide on neuroexocytosis, another set of experiments was performed using an intracellular electrode to record the evoked junctional potentials (EJP) from the mouse diaphragm. Fig. 2C shows that h-JMS inhibits very rapidly the EJPat the mouse NMJ.

As shown in Fig. 1, the juxtamembrane segment is conserved among species and, therefore, we tested the effect of the h-JMS on the *D. melanogaster* third instar larva NMJ. Fig. 2D shows that this peptide is a highly effective inhibitor of neurotransmitter (glutamate) release at the insect NMJ reinforcing the suggestion that the JMS of Syt plays a major role in neuroexocytosis.

**Fig. 3:**
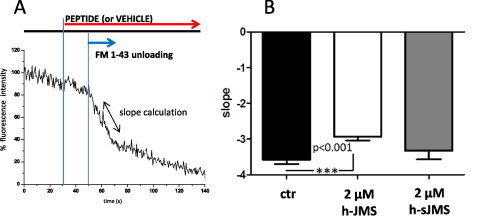
Effect of synaptotagmin h-JMS on the neuroexocytosis of primary spinal cord motor neurons. Rat primary motor neurons (7–12 DIV) were loaded with 7 μM FM 1–43 by electrical field stimulation. After washing, neurons were exposed to different concentrations of peptide or vehicle; another electric stimulation induces the unloading phase, which was monitored by fluorescence microscopy, as described in the scheme at the top of panel A). Images were analyzed selecting fluorescent ROI (15–20 for each experiments) along neurites (with fixed dimensions of 1.6 μm × 1.6 μm remaining immobile during experimental time). The fluorescence intensity was plotted for each region and the slope corresponding to the unloading phase was calculated. A representative trace, corresponding to one ROI is shown as example. B) The graph represents the average slope of data obtained from four different cell preparations; 2 μM peptide *n* = 84; 2 μM scrambled peptide *n* = 42; control *n* = 84.

This process can be assayed directly by imaging the release of appropriate fluorescent dyes. Using rat primary spinal cord motor neurons loaded with FM 1–43, we compared the release of the dye in the presence or absence of the h-JMS peptide. As schematized in Fig. 3A, for each experiment, regions corresponding to the synaptic sites were selected, and the fluorescence intensity measured during the unloading phase; the slope that characterizes this phenomenon (i.e., the speed of the exocytic event) was chosen to compare different experiments. Using this experimental approach, we observed a significant inhibition of FM 1–43 release by the h-JMS peptide, but not by the scrambled form (Fig 3B), further supporting the indication that the juxtamembrane segment of Syt is involved in neuroexocytosis.

### 2.2 The h-JMS peptide preferentially localizes at the presynaptic membrane

To visualize the site(s) of binding and action of the h-JMS peptide, we prepared a fluorescent derivative (Fig. 1F, h-FJMS), using an additional lysine residue to covalently bind the peptide both to the hexyl moiety and to a fluorescent moiety. This peptide was tested in the hemidiaphragm preparation and found to inhibit neurotransmitter release (not shown).

**Fig. 4:**
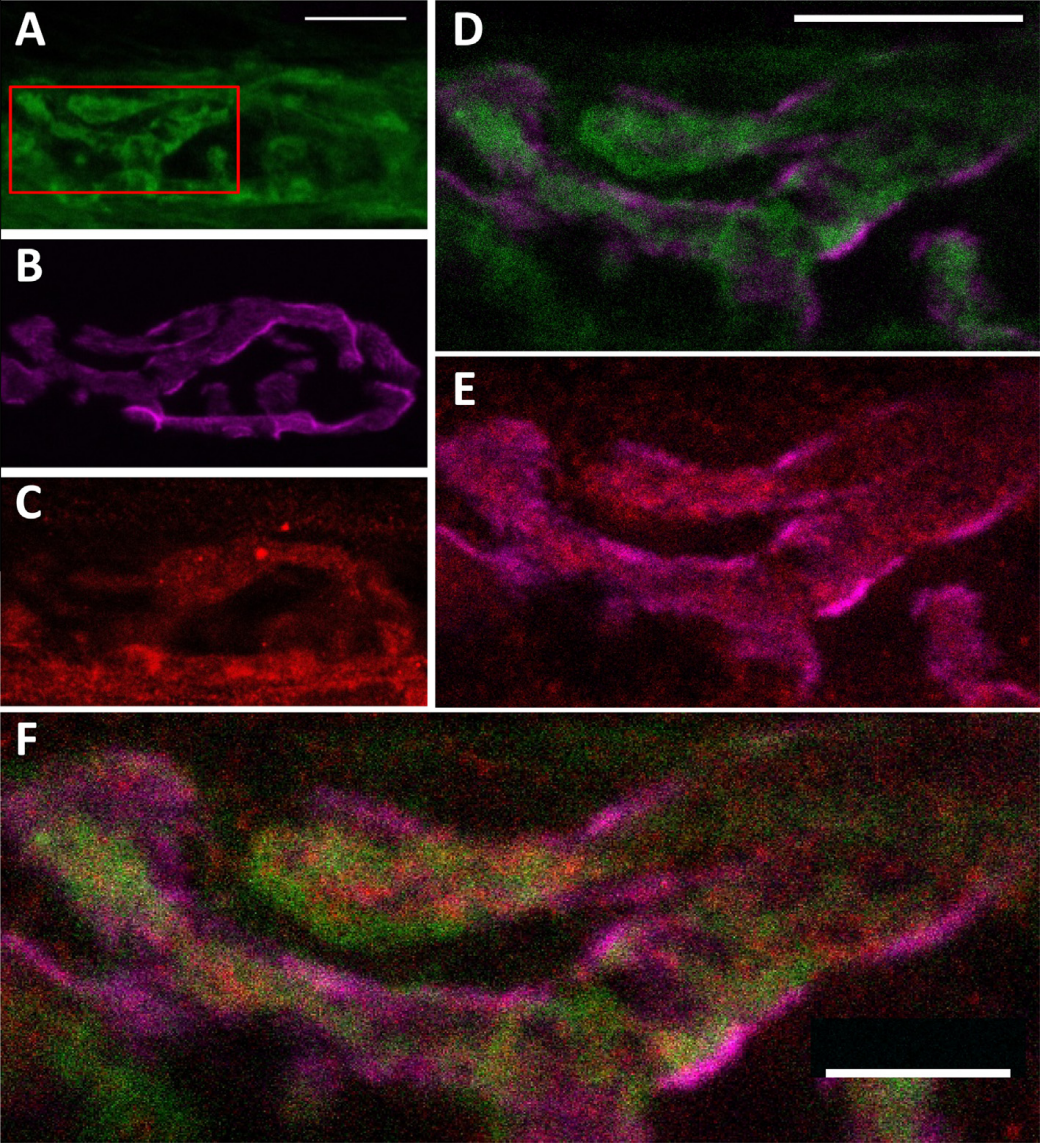
Localization of the fluorescent peptide h-FJMS at the mouse hemidiaphragm neuromuscular junction. The mouse hemidiaphragm neuromuscular junction preparation was fixed when paralysis caused by 200 μM h-FJMS peptide was attained and immunostained. (A) Fluorescent peptide (green); (B) α-Btx (purple, post synaptic marker); (C) Basson (red, pre-synaptic active zone marker); (D and E) merged images of the red zone in A. (F) merge of the three markers used, indicating a membrane presynaptic localization of the fluorescent peptide. Scale bar: 10 μm in A–E; 5 μm in F.

**Fig. 5:**
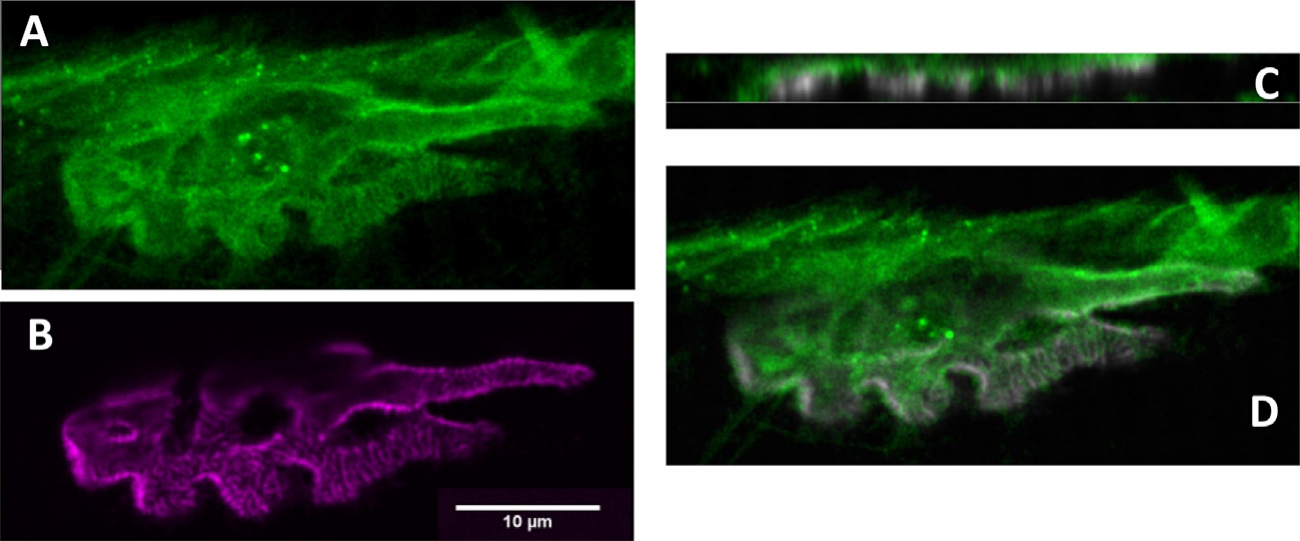
Localization of the h-FJMS peptide at the neuromuscular junction. Hemidiaphragm fixed at paralysis and immunostained after treatment with 200 μM FITC-labeled hexyl-juxtamembrane peptide (h-FJMS). (A) fluorescent peptide (green); (B) α-BTX (purple, post synaptic marker); (C) frontal and (D) lateral merged images, showing the presynaptic localization of h-FJMS (green).

**Fig. 6:**
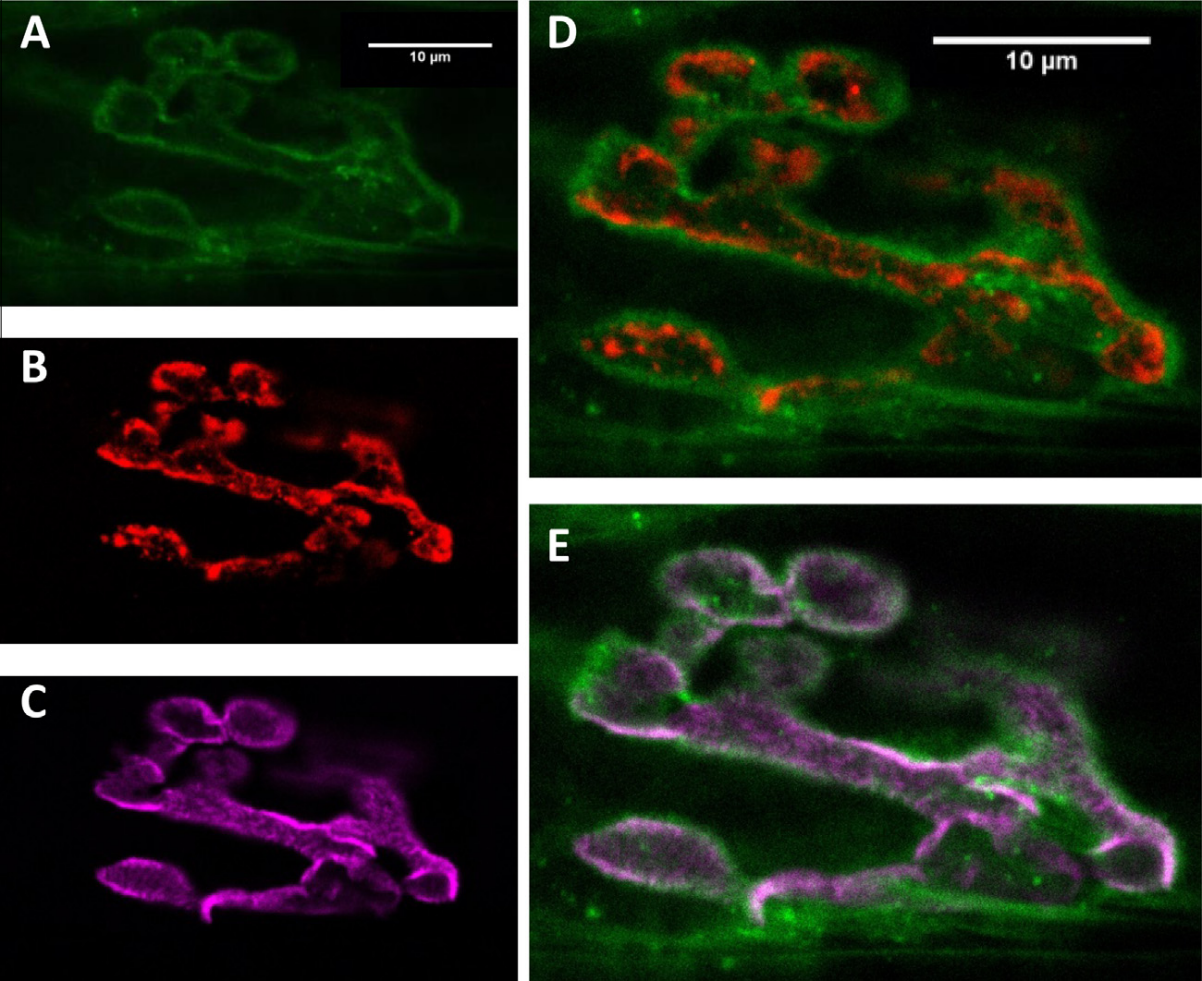
Localization of the h-FJMS peptide and synaptic vesicles at the NMJ. Hemidiaphragm fixed at paralysis and immunostained after treatment with 200 μM h-FJMS peptide. (A) h-FJMS peptide (green); (B) anti VAChT (red); (C) α-BTX (purple, post synaptic marker); (D and E) merged images.

Fig. 4 shows that, in the mouse hemidiaphragm NMJ preparation, the h-FJMS staining is largely confined to the presynaptic membrane and the periplasmic area as indicated by the substantial overlap with the staining of the presynaptic membrane marker Bassoon and by its distribution with respect to the post-synaptic marker α-bungarotoxin. This is particularly evident in the lateral section (Fig. 5). Out of line with these results, unexpectedly, there is a comparatively lower staining by the h-FJMS peptide of the SVs, which are largely localized inside the motor axon terminal, as documented by comparison with the staining of an antibody specific for the vesicular acetylcholine transporter (Fig. 6). Taken together these data suggest that a large part of the fluorescent peptide inserts into the presynaptic membrane. This is in agreement with the fact that similar cationic peptides of the C2 domains of Syt bind to the PM via interaction with anionic phospholipids [16,17,38–40].

### 2.3 The h-JMS peptide binds to anionic phospholipids independently of Ca^2+^

**Fig. 7:**
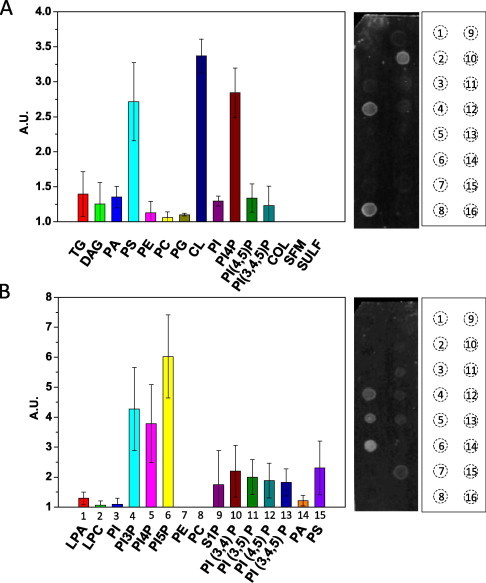
Lipid binding of the fluorescent h-JMS peptide. PIP-strip membranes spotted by manufacturer with the different phospholipids were incubated with 5 μg/ml peptide for 75 m, washed and the fluorescence was measured. (A) general lipid binding: TG, triglycerol; DAG: diacylglycerol; PA: phosphatidic acid; PS: phosphatidylserine; PC: phosphatidylcholine; PG: phosphatidylglycerol; CL: cardiolipin; PI: phosphatidylinositol; PI4P: phosphatidylinositol-4-phosphate; PI(4,5)P: phosphatidylinositol 4,5-bisphosphate, PI(3,4,5): phosphatidylinositol 3,4,5-trisphosphate; COL: cholesterol; SFM: sphingomyelin; SULF: sulfatide. (B) Phosphoinositides binding: LPA: lysophosphatidic acid; LPC: lysophosphatidylserine; PI: phosphatidylinositol; PI3P: phosphatidylinositol 3-phosphate, PI4P: phosphatidylinositol 4-phosphate; PI5P: phosphatidylinositol 5-phosphate; PE: phosphatidylethanolamine; PC: phosphatidylcholine; PI(3,4)P: phosphatidylinositol 3,4-bisphosphate; PI(3,5)P: phosphatidylinositol 3,5-bisphosphate; PI(4,5)P: phosphatidylinositol 4,5-bisphosphate; PI(3,4,5)P: phosphatidylinositol 3,4,5-trisphosphate; PA: phosphatidic acid; PS: phosphatidylserine. In both panels, bars represent the fluorescence for each lipid spot normalized to the blank. A scheme of the PIP-strip membrane used is shown on the right, and the corresponding number is reported below the graphs. Data are the mean of three independent experiments ± s.d. The images are a representative strip for each experiment.

Therefore, we assayed the binding of the fluorescent peptide h-FJMS to a variety of anionic phospholipids. Fig. 7 shows that the h-FJMS indeed binds anionic phospholipids, including phosphatidylserine and phosphatidylinositides (PIs), which are enriched in the SV membrane and on the cytosolic face of the PM [39,41]. However, in contrast to the Ca^2+^-dependent binding of PIP_2_ to the C2 domains, the lower panel of Fig. 7 documents that the h-FJMS peptide binds mono-phosphorylated phosphoinositides independently of the presence of Ca^2+^. In addition, we found little or no binding to the other classes of phospholipids tested, with exception of cardiolipin, which, however, contains two phosphate groups in its molecule. This lipid binding pattern is similar to the one found previously using the sumoylated Syt 1 peptide 80–96 [42]. Similar anionic lipid binding by a polybasic juxtamembrane segment were found in syntaxin [43] and in VAMP [44]. Therefore, it is very unlikely that the present finding is an artefact resulting from the presence of the hexyl and the fluorescent moieties. As PS and PIs are present in the cytoplasmic side of the presynaptic membrane and on the SV membrane, this binding may have a great significance in the SV–PM interaction. The possible importance of the cluster of basic residues in the juxtamembrane segment of the two SNARE proteins VAMP and syntaxin in the interaction with anionic phospholipids was proposed right after the report of the structure of the SNARE complex [45] as well as the electrostatic interaction among proteins and plasma membrane phosphoinositides [46]. This will be discussed below for the case of the vesicle protein synaptotagmin reported here together with a possible novel role of the juxtamembrane segment of this protein in synaptic vesicle membrane fusion.

## 3 Discussion

The main finding of the present work is that peptides corresponding to the segment 80–98 of Syt 1 (JMS) which is responsible for Ca^2+^ triggered neuroexocytosis, cross the plasma membrane and strongly inhibit neuroexocytosis, most likely by competing with synaptotagmins at the mouse and *D. melanogaster* neuromuscular junction. The capability of highly cationic peptides to cross the plasma membrane and enter cells is well documented though the mechanism(s) and subcellular site(s) of entry are ill defined [32–34]. The powerful inhibition of neuroexocytosis by JMS peptides, in all likelihood, results from its ability to compete for a yet undisclosed essential interaction that Syt displays *in vivo* with an unknown partner via its juxtamembrane segment. That this interaction has to occur close to the cytosolic surface of the SV membrane or the PM membrane, or both, is suggested not only by the location of JMS with respect to the TM domain of Syt, but also by the much higher inhibitory activity of the JMS hexyl derivative that partition in the membrane via its hydrocarbon chain. Accordingly, we have tested the interaction of JMS with phospholipids and have found a defined interaction of JMS with anionic phospholipids, including PS and phosphorylated derivatives of phosphatidylinositols, which are present on the cytosolic monolayers of both SV and PM membranes. Such lipid interaction occurs independently of Ca^2+^ in the medium, suggesting that the JMS segment of Syt may interact *in vivo* both with the SV membrane (*cis* interaction) and with the PM (*trans* interaction). On the basis of this finding, we would like to propose that the JMS segment is defined as a domain of Syt endowed with the function of Ca^2+^-independent anionic phospholipid binding. To localize the site of action of the hexyl derivative JMS peptides, we have used a fluorescein derivative of the hexyl-JMS domain (h-FJMS of Fig. 1). Unexpectedly, h-FJMS was found to localize more on the presynaptic membrane than on the SV present inside the nerve terminal. The presynaptic labeling of h-FJMS is remarkable and indicates that its partitioning within the neuromuscular junction is rather specific, providing a further indication of the validity of the results of inhibition of neuroexocytosis. There is little staining of h-FJMS of the cytosol of the NMJ which tends to exclude, in the present case, an indirect effect of the JMS peptide via actin-remodeling similar to the one found by Johnsson and Karlsson [42] in cultured neurons. In fact, neurons in culture bear little resemblance to the *ex vivo* NMJ preparations used here, which include muscle and peripheral Schwann cells.

**Fig. 8:**
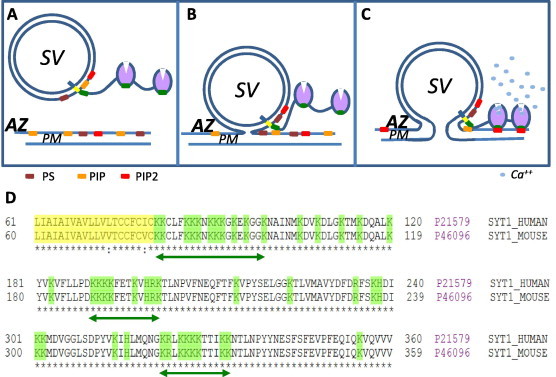
A possible role of the synaptotagmin juxtamembrane segment in synaptic vesicle–plasma membrane interaction and hemifusion. (A) synaptic vesicle (SV) near the presynaptic membrane (PM) before fusion; green segments represent the three polybasic sequences in synaptotagmin, localized in the juxtamembrane segment and in the two C2 domains capable of anionic phospholipid binding; (B) interaction of the juxtamembrane segment of synaptotagmin residing on a hemifused SV with anionic lipids of the cytosolic surface of the PM (preferentially mono-phosphorylated phosphoinositides, orange) could favor the close apposition of the two membranes with hemifusion. AZ: active zone. (C) The rise of calcium (light blue dots) consequent to the opening of calcium channels nearby (not shown) will then cause the C2 domains conformational change allowing their interaction with PIP2 (red) and the complete membrane fusion. (D) Sequence of the three polybasic peptide segment in mouse and human synaptotagmin 1.

Clearly, the localization of h-FJMS does not reflect that of the JMS domain as part of the entire Syt molecule *in vivo* as Syt is an integral membrane protein of SV and the JMS peptide is a small molecule. However, this result may be taken as an indication that JMS has a stronger affinity for the phospholipids of the PM with respect to those of the SV, though the molecular basis of this preference are not known. It is possible that the cytosolic face of the plasma membrane contains clusters of phosphoinositides and that this feature favors a strong interaction with cluster of positive residues of proteins as discussed in [46] and references cited there in. Translated to the *in vivo* situation, the localization of h-FJMS may indicate that JMS mediates cis-interactions with its own membrane, but, when SV become close enough to the PM, such as in the case of the docked vesicles [47], then JMS can switch to a trans-interaction with the cytosolic face of the PM where PIs are localized, as schematized in Fig. 8. Such a possibility posited here for the first time is intriguing and adds to the well defined cis and trans interactions of the C2 domains of Syt in a Ca^2+^-dependent mode [2,16,17,38,40,48]. The interaction of the Syt of docked vesicles with anionic phospholipids of the cytosolic monolayer of the PM would implicate a very close apposition of the two membranes, thus promoting the possibility of hemifusion of the vesicle membrane with the plasma membrane (Fig. 6). There is an almost general consensus that membrane hemifusion is an essential step preliminary to fast exocytosis [2,31,49,50] and the possibility that the juxtamembrane domain of synaptotagmin is involved in membrane hemifusion deserves all the attention and it is amenable of testing by electron microscopy and biophysical methods.

## 4 Materials and methods

### 4.1 Antibodies

Rabbit polyclonal anti vesicular acetylcholine transporter (VAChT) and mouse monoclonal anti Basson are from Synaptic System (Goettingen, Germany); α-Bungarotoxin, Alexa Fluor® 555 conjugate and secondary anti mouse-rabbit Alexa Fluor® 647 conjugate antibodies are from Molecular Probes, Invitrogen (Carlsbad, CA).

### 4.2 Peptides synthesis

The synthetic peptide encompassing the synaptotagmin sequence (80–98) and its derivatives were prepared by solid phase peptide synthesis using a multiple peptide synthesizer (SyroII, MultiSynTech GmbH) on Rink Amide MBHA resin (Novabiochem, Bad Soden, Germany). The fluoren-9-ylmethoxycarbonyl (Fmoc) strategy [51] was used throughout the peptide chain assembly, using O-(7-Azabenzotriazol-1-yl)-N,N,N′,N′-tetramethyluronium hexafluorophosphate (HATU) (ChemPep, Wellington, Fl USA) as coupling reagent [52]. The side-chain protected amino acid building blocks used were: N-α-Fmoc-γ-tert-butyl-l-glutamic acid, N-α-Fmoc-Nε-(tert-butyloxycarbonyl)-l-lysine, N-α-Fmoc-Nε-(4-methyltrityl)-l-lysine, N-α-Fmoc-S-trityl-cysteine, N-ε-(tert-butyloxycarbonyl)-aminohexanoic acid. Fluorescent labeling was performed during the synthesis with 5(6)-carboxyfluorescein (FAM). For this purpose the peptide (see peptide c, Fig. 1) was synthesized with an additional N(epsilon) protected lysine with the orthogonal methyltrityl (Mtt) group. The Mtt group was selectively removed by treatment with a 10% acetic acid in dichloromethane followed by a second treatment with 1% trifluoroacetic acid, 10% trifluoroethanol in dichloromethane. Then, the fluorescent probe (FAM) was pre-activated with DIC (N,N′-diisopropylcarbodiimide) and HOAt (1-Hydroxy-7-azabenzotriazole) (ChemPep, Wellington, Fl USA) for 10 min and added to the peptidyl resin. The reaction proceeded for 2 h under stirring. Cleavage of the peptides was performed by incubating the peptidyl resins with trifluoroacetic acid/H_2_O/thioanisole/ethanedithiol/phenol (10 ml/0.5 ml/0.5 ml/0.25 ml/750 mg) for 2.5 h. Crude peptides were purified by a preparative reverse phase HPLC. Molecular masses of the peptide were confirmed by mass spectroscopy on a MALDI TOF-TOF using a Applied Biosystems 4800 mass spectrometer.

### 4.3 Cell cultures

Primary rat spinal cord motor neuron (MNs) were isolated from Sprague-Dawley rat embryos (embryonic day 14) and cultured following previously described protocols [53]. All experiments were performed using MNs differentiated for 6–12 days.

### 4.4 Mouse phrenic nerve-hemidiaphragm preparations

All experimental procedures were performed in accordance with the Italian guidelines, law n. 116/1992 and were approved by the Animal Ethical Committee of our University.

Mouse phrenic nerve hemidiaphragms were isolated from male CD-1 mice weighing about 20–25 g and mounted in 2–3 ml oxygenated (95% O_2_, 5% CO_2_) Krebs–Ringer solution (139 mM NaCl, 12 mM NaHCO_3_, 4 mM KCl, 2 mM CaCl_2_, 1 mM MgCl_2_, 1 mM KH_2_PO_4_ and 11 mM glucose, pH 7.4). Two innervated hemidiaphragm preparations were isolated from each animal.

### 4.5 Muscle twitch measurements

The phrenic nerve was stimulated via two ring platinum electrodes with supramaximal stimuli of 10 V amplitude and 0.1 millisecond pulse duration, with a frequency of 0.1 Hz (Stimulator 6002, Harvard Apparatus, Massachusetts, USA). Muscle contraction was monitored with an isometric transducer (Harvard Apparatus); data were recorded and analyzed by the i-WORX 118 system (Harvard Apparatus). The amplitude of twitch was calculated as a difference from basal muscular tension and the mean of peak value measured after stimulation.

Muscles were stretched to the optimal length for twitch tension and the muscle twitch allowed to stabilize for at least 20 min at 37 °C. In control experiments the amplitude of muscle contraction under this type of stimulation was constant for at least 8 h. The mean twitch value measured during the last 5–10 min before the experiment was taken as 100% in order to normalize the data.

Peptides at the indicated concentrations were added to the nerve-muscle preparations in the minimal volume of buffer.

### 4.6 Muscle electrophysiology

Experiments in mouse NMJ were performed on mouse phrenic nerve hemidiaphragm preparations, pinned on silicone-coated surface (Dow Corning, Germany), maintained in oxygenated Krebs–Ringer solution, as described previously [54]. A 3 μM final concentration of μ-Conotoxin GIIIB (Alomone Labs, Israel) was added to the bath to block muscle action potentials. Peptides at the indicated concentrations were added to the nerve-muscle preparations in the minimal volume of buffer. Evoked Junction Potentials (EJPs) were intracellularly recorded, in current-clamp mode, in single muscle fibers using glass microelectrodes following stimulation of phrenic nerve stump, at 0.5 Hz. Phrenic nerve stump was stimulated using a suction microelectrode connected to a stimulator (Grass S88, USA) through a Stimulus Isolation Unit (SIU5, Grass, USA). Recorded signals were offline analyzed with an appropriate software (Pclamp, Molecular Devices, USA). Relative EJPs amplitude was calculated with respect to the average of the first 10 evoked EJPs recorded in physiological conditions.

### 4.7 *D. melanogaster* Electrophysiology

Experiments using the *Drosophila* NMJ were performed at room temperature on third instar larval body wall preparations dissected in Ca^2+^-free HL3 saline and pinned on the silicone-coated surface (Dow Corning, Germany) as described previously [10]. After dissection Ca^2+^ free HL3 saline was replaced with 1 mM Ca^2+^ HL3. Peptides at the indicated concentrations were added to the nerve-muscle preparations in the minimal volume of buffer. Electrophysiological recordings were performed on fibers 6 or 7 of abdominal segment 3 or 4 using intracellular glass microelectrodes (WPI, Germany). Fibers with a membrane resting potential lower than −60 mV were rejected. Recorded signals were offline analyzed with an appropriate software (Pclamp, Molecular Devices, USA). Relative EJPs amplitude was calculated with respect to the average of the first 10 EJPs recorded in physiological conditions.

### 4.8 FM 1–43 unloading

Rat primary spinal cord motor neurons (7–12 DIV) were loaded with 7 μM FM 1–43 (Molecular Probes, Invitrogen, Carlsbad, CA) by electrical field stimulation (20 s at 20 Hz, 400 Action Potential, AP). For each culture, a preliminary test with the calcium binding dye Fluo 4 (Molecular Probes, Invitrogen, Carlsbad, CA) was performed, to verify that the voltage applied (15–18 V) induces a transient Ca^2+^ influx without damaging cells. After FM 1–43 wash, neurons were exposed to different concentrations of peptide or vehicle; the unloading phase was induced with another electric stimulation (400 AP) and monitored by fluorescence microscopy. Images were acquired by a Nikon TE2000E stage, and analyzed with the ImageJ (http://rsbweb.nih.gov/ij/). For each experiment, 15–20 ROIs were selected with fixed area and dimensions (1.6 μm × 1.6 μm) and remaining immobile during experimental time. The normalized fluorescence intensity for each region was corrected subtracting the residual fluorescence at the end of the experiment; for the data of the initial phase of unloading (first 20 s) a linear fitting was performed and the corresponding slope calculated. ROIs with fitting *R* square below 0.8 were discarded. The average slope was taken as indicator of the exocytosis speed.

### 4.9 Protein lipid overlay

“PIP Strips” (nitrocellulose membranes prespotted with defined lipids) were purchased from Echelon Biosciences (Salt Lake City, UT) and binding overlay experiments were carried out according to the manufacturer’s protocol. Briefly, the nitrocellulose was saturated with TrisCl 50 mM, NaCl 150 mM pH 6.8–7, Tween 20 0.5% (TBS-T), BSA FA free 3% over night at 4 °C, and then incubated with 5 μg/ml h-FJMS in the same solution for 2 h at RT. The nitrocellulose was washed extensively with TBS-T, and the bound protein was detected by fluorescence assay. Images were taken using a Kodak Image Station 4000 MM Pro. Integrated densities at each lipid spot (three replicates) were measured with Image J and normalized to the blank value for each treatment.

### 4.10 Fluorescent labeling and Immunohistochemistry

Hemidiaphragms from twitch experiments with the fluorescent peptide (h-FJMS) were washed and fixed (2 h at room temperature) with 4% paraformaldehyde, 20% sucrose, washed with PBS and 50 mM NH_4_Cl for 10 min. Muscles were treated with 2% bovine serum albumin, 0.25% porcine skin gelatin, 0.2% glycine, and 15% goat serum in PBS (blocking solution) plus 0.5% Triton X-100 to permeabilize. Muscles were then incubated with primary antibodies in blocking solution for 24 h at 4 °C; after washing, secondary fluorescent antibody and α-bungarotoxin Alexa-555 were added and incubated for 2 h at room temperature. Images were collected from whole-mount preparations in Mowiol (Sigma, St. Louis, MO) with Leica SP-5 confocal microscope.

## Competing interests

The authors declare no competing interests

## Authors contribution

P.C. and C.M. conceived the project; N.D. and O.M. synthesized and purified all the peptides, P.C., M.S. and A.M. performed the experiments and all authors analyzed the data, C.M. wrote the paper with contributions of all authors.
